# A Social Media Campaign (#datasaveslives) to Promote the Benefits of Using Health Data for Research Purposes: Mixed Methods Analysis

**DOI:** 10.2196/16348

**Published:** 2021-02-16

**Authors:** Lamiece Hassan, Goran Nenadic, Mary Patricia Tully

**Affiliations:** 1 Division of Informatics, Imaging and Data Sciences The University of Manchester Manchester United Kingdom; 2 Department of Computer Science The University of Manchester Manchester United Kingdom; 3 Division of Pharmacy and Optometry The University of Manchester Manchester United Kingdom

**Keywords:** social media, public engagement, social network analysis, medical research

## Abstract

**Background:**

Social media provides the potential to engage a wide audience about scientific research, including the public. However, little empirical research exists to guide health scientists regarding what works and how to optimize impact. We examined the social media campaign #datasaveslives established in 2014 to highlight positive examples of the use and reuse of health data in research.

**Objective:**

This study aims to examine how the #datasaveslives hashtag was used on social media, how often, and by whom; thus, we aim to provide insights into the impact of a major social media campaign in the UK health informatics research community and further afield.

**Methods:**

We analyzed all publicly available posts (tweets) that included the hashtag #datasaveslives (N=13,895) on the microblogging platform Twitter between September 1, 2016, and August 31, 2017. Using a combination of qualitative and quantitative analyses, we determined the frequency and purpose of tweets. Social network analysis was used to analyze and visualize tweet sharing (*retweet*) networks among hashtag users.

**Results:**

Overall, we found 4175 original posts and 9720 retweets featuring #datasaveslives by 3649 unique Twitter users. In total, 66.01% (2756/4175) of the original posts were retweeted at least once. Higher frequencies of tweets were observed during the weeks of prominent policy publications, popular conferences, and public engagement events. Cluster analysis based on retweet relationships revealed an interconnected series of groups of #datasaveslives users in academia, health services and policy, and charities and patient networks. Thematic analysis of tweets showed that #datasaveslives was used for a broader range of purposes than indexing information, including event reporting, encouraging participation and action, and showing personal support for data sharing.

**Conclusions:**

This study shows that a hashtag-based social media campaign was effective in encouraging a wide audience of stakeholders to disseminate positive examples of health research. Furthermore, the findings suggest that the campaign supported community building and bridging practices within and between the interdisciplinary sectors related to the field of health data science and encouraged individuals to demonstrate personal support for sharing health data.

## Introduction

### Social Media Use by Academics

Social media platforms such as Twitter, LinkedIn, and Facebook have changed the way scientists interact with others, both socially and professionally. Although the specifics may vary between individuals, platforms, and scientific disciplines [1], common scholarly purposes for using social media among academics include discovering peers and enhancing collaboration, sharing links or citations to their own or others work, communicating the proceedings of conferences and meetings, raising their own profiles, engaging in discussions and keeping up to date with scholarly work, answering questions and solving problems, and discovering job opportunities [2-13].

There is also a growing interest in using social media to engage a wider audience about scientific research, including the public [6,14,15]. A recent scoping review of health scientists’ strategies by Fontaine et al [16] identified 9 types of science communication strategies used by health scientists, directed at areas such as content, engagement, intention, presentation, and statistics. However, the same review concluded that empirical studies in this field were lacking, representing a missed opportunity to understand how to optimize science communication strategies.

### A Social Media Campaign for Health Informatics Research: #datasaveslives

The social media campaign #datasaveslives [17] was established in 2014 by the Northern England branch of the Farr Institute for Health Informatics Research, a publicly funded, UK-wide research collaboration involving academic institutions and health partners. The campaign started with a simple goal: to promote the positive use of data in health research on social media. A select group of academic organizations belonging to, or affiliated with, the Farr Institute subsequently formally adopted #datasaveslives as part of their communications and stakeholder engagement strategies [18]. These supporters then encouraged a wider audience of people who supported health data research to use the hashtag #datasaveslives on social media sites (primarily Twitter) to index and share examples that demonstrate how health data from patient records and other sources could be used to create public health benefits. The second objective is to spark interest and dialog about using health data for research purposes among wider audiences, including patients, members of the public, health care professionals, and policy makers.

### About Twitter

Twitter is a popular microblogging social media platform founded in 2006 [19]. It allows users to post short messages (previously 140 characters, more recently extended to 280) known as *tweets*, which may also include URL links, multimedia content (eg, images or videos) and/or references to other users (signified using the *@* symbol, plus a username). Hashtags may also be used by assigning the *#* character to a term of their choice; this is a useful way of indexing and searching for tweets on a similar topic. Users can view and engage with tweets in a number of ways, including liking, replying to, and sharing (*retweeting*) others’ posts. They can also follow others to *subscribe* to see their tweets. Tweets are public by default, although users can change their settings at any time to restrict their visibility to their Twitter followers. Users can also choose to write a short description about themselves (known as a *bio*) and add their location.

### Study Aim

The aim of this study is to examine how the #datasaveslives hashtag has been used on Twitter in the context of the use of data in health research and by whom. The analysis will determine how often the hashtag has been used and shared and examine the content posted alongside the hashtag to determine the range of purposes for its use. This will provide insights into the strategic use of social media campaigns by academics and explore their potential for encouraging wider dialog within and between scientific communities and broader audiences.

Specifically, the following objectives (and research questions in brackets) were defined:

1. Determine the frequency of tweets and retweets featuring the hashtag #datasaveslives, including the most frequently shared tweets (how often was #datasaveslives tweeted?).
2. Characterize the range of stakeholder groups that use and share #datasaveslives and visualize retweet relationships between users (who tweets #datasaveslives and how were tweets shared between users?).
3. Identify and explore the different purposes that people used #datasaveslives for when tweeting (what did people use #datasaveslives to tweet about?).

## Methods

### Design and Objectives

We used a mixed methods design, combining elements of descriptive statistics, social network analysis, and qualitative research. This approach, which used a combination of qualitative and quantitative analysis, was adopted to allow a richer analysis of Twitter posts, over and above what could be achieved by available social media analytics tools.

### Data Set, Variables, and Definitions

The data set comprised all publicly available tweets (N=13,895) that included the hashtag #datasaveslives posted between September 1, 2016, and August 31, 2017. This year was selected because it was perceived to represent a peak in campaign activity, thereby providing a sufficiently large and diverse sample of tweets for analysis. These were procured from Twitter’s historical data service in January 2018.

The following variables pertaining to the tweet text and metadata associated with the tweet were retained for the analysis: tweet ID, tweet text (*body*), a list of hashtags included in tweets, number of retweets, and date posted (recoded into day, month, and year).

Twitter classifies each tweet as either an original *post* or a *share*. Posts were defined as tweets where the user either created a new tweet with their own original text or where a user shared another user’s tweet and added new text to accompany it (*quote* tweets). Shares (more commonly referred to as *retweets*) referred to cases when the user had shared a post created by another user with their followers without changing or adding new text. In all cases, tweets were only included if they referenced #datasaveslives somewhere in the body of the tweet, whether in the shared text or the text newly added by the user.

Where available, we also retained the following data pertaining to individual users who posted tweets, specifically: username, bio (optional self-written text about the user in 160 characters or less), friend count (users they had elected to follow), and follower count (users who had elected to follow them).

For analysis purposes, we defined official supporters as the 6 user accounts belonging to the sites of the Farr Institute and the Connected Health Cities (CHC) programme, all of whom adopted #datasaveslives as part of their formal strategies (@FarrInstitute, @CHCNorth, @HeRC_Farr, @FarrScotland, @FarrCIPHER, and @FarrLondon).

### Data Preprocessing and Analysis

Historical Twitter data were preprocessed using Python (version 3.7.2). Briefly, the *pandas* Python library was used to convert data from a JavaScript Object Notation (JSON) format into a two-dimensional data frame for cleaning, recoding, and validation tasks in preparation for data analysis.

Statistical analyses were completed using RStudio (version 1.1.456). To address objective 1, descriptive statistics were used to determine weekly and monthly frequencies of tweets featuring #datasaveslives and percentages of the most commonly shared tweets (retweets). Pearson R was used to determine the associations between weekly counts for posts and retweets. For the most commonly shared retweets, the total potential reach was estimated by summing the follower count for every user who shared the tweet.

To address objective 2, users were grouped according to tweet frequency, and their characteristics were analyzed in terms of median counts for followers, friends, and posts. Gephi (version 0.9.2), a social network analysis tool, was used to analyze and visualize relationships between users of #datasaveslives. We focused on the retweet network as a way of understanding the sharing practices and underlying network structures between users. Statistics about the overall network and individual vertices were generated based on who retweeted whom, including clustering coefficients and measures of centrality. These were used to produce an undirected network graph visualizing the connections (edges) between users (vertices). To detect communities and calculate modularity, we used the Louvain method for community detection, which has been shown to outperform similar modularity methods in terms of speed and efficiency [20]. The graph was laid out using the Force Atlas layout algorithm. Common words used in user bios and tweet texts were also identified for each cluster (excluding commonly used words, eg, *and*, *or*, *views*).

To address objective 3, thematic analysis [21] was used to analyze the textual content of tweets featuring #datasaveslives qualitatively. Owing to the large size of the data set, it was not deemed practical or necessary to read and code all tweets. All original posts accompanying the hashtag were imported into NVivo 12 [22] for analysis. After reading a convenience sample of tweets (the first 200 tweets in date order), we defined an initial coding structure, covering the range of purposes tweets appeared to be used for. All original posts were sorted using the random number generator function in Microsoft Excel and then reviewed, coded, recoded, and collated into key themes in an iterative fashion by LH. Tweets were coded until saturation occurred, that is, until no substantially new themes were found. Approximately 1000 tweets were manually reviewed in total. The final set of themes was decided upon following a discussion between the authors.

### Ethics and Governance

Data were collected and processed in line with Twitter’s terms and conditions. As this information was nonsensitive and already in the public domain, formal ethical approvals were not required to complete the project.

On advice from our university’s research ethics office and in line with wider social media research guidelines [23], we took the following measures to protect Twitter users’ privacy and confidentiality expectations: first, only tweets of users with accounts set to *public* were included in the analysis. Second, we gained permission to quote verbatim posts by individual users who were not clearly part of identifiable public groups or bodies or tweeting in an official capacity (eg, government organizations, university departments, heads of department). During the course of identifying popular tweets, we discovered that some posts or accounts had subsequently been deleted by users following the time of data collection; in such cases, tweets were not quoted although they were retained for the purposes of aggregated quantitative and qualitative analyses.

## Results

### How Often Was #datasaveslives Tweeted?

**Figure 1:**
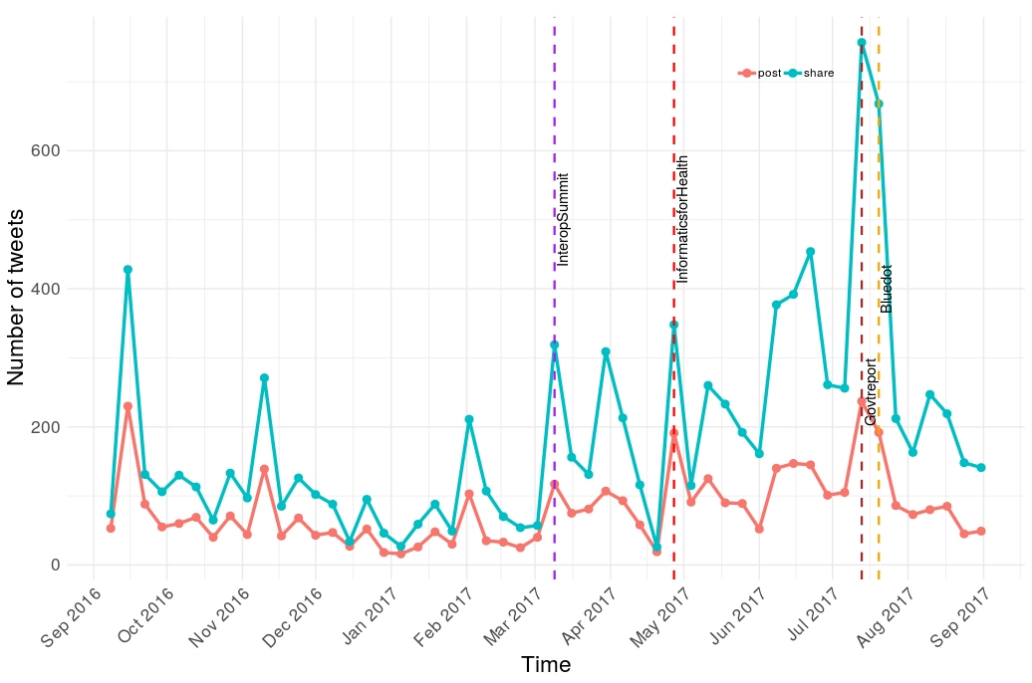
Tweet frequency over time by tweet type.

During the observation period, there were 13,895 tweets containing #datasaveslives (Figure 1). Overall, 30.05% (4175/13,895) were original posts and 69.95% (9720/13,895) were retweets. Among the original posts, 34.80% (1453/4175) were quote tweets. The mean number of total weekly tweets was 267.21 (SD 200.06), although this varied substantially (range 43-994). There was a strong positive correlation between weekly counts for posts and retweets (*r*=0.927, *df*=50; *P*<.001).

The highest number of tweets was observed during the week commencing July 6, 2017 (237 posts and 757 retweets), during which the UK government published a response [24] to a national review of security, consent processes, and opt-outs relevant to health data [25]. During the same week, there were also tweets about public engagement activities at high-profile cultural festivals in Cheshire (Bluedot Festival, England) and Edinburgh (Edinburgh Festival Fringe, Scotland). There were also high frequencies of tweets from official supporters during the week beginning April 20 (week 34), when there was a health informatics conference (Informatics for Health) hosted in Manchester in England.

Overall, 6 of the 10 most frequently shared tweets were from accounts associated with organizations, networks, or events (Table 1); only 1 originated from the account of an official supporter (@HerC_Farr). There was a modest, though significant, positive correlation between retweet count and follower count (*r*=0.214, *df*=4173; *P*<.001).

**Table 1:** The top 10 most frequently shared tweets.

Rank	Tweet^a^	Username and bio	Retweets, n	Group^b^	Total potential follower reach, n^c^
1	“Without data, this wouldn’t be possible. We welcome the Govt’s response to @NDGoffice review #DataSavesLives”	@NHSDigital; Information and technology for better health and care	58	7	274,311
2	“#DataSavesLives Our open letter from charities following the Government’s response to the Caldicott Review”	@wellcometrust; We’re a charitable foundation that exists to improve health for everyone. We support thousands of scientists & researchers, spark debate & take on big problems	51	3	206,171
3	Not available. Tweet deleted by the user	Bio not available	50	N/A^d^	N/A
4	“Remembering Alan Turing today, on his anniversary. An incredible scientist and human being, and an original believer in #datasaveslives”	@HeRC_Farr; An academic, NHS & Industry Partnership: Harnessing health data for patient and public benefit. #datasaveslives	41	1	52,544
5	“Better use of data means you don’t have to tell your story again and again to doctors and nurses #DataSavesLives”	@NHSEngland; Health and high quality care for all, now and for future generations	40	10	212,478
6	“Using patient data is vital to improve health+care for us all #datasaveslives”	@NMRPerrin; Leading new Understanding Patient Data initiative. Interested in all things data, with a bit of science policy on the side	38	3	35,532
7	“Come + work with me! Understanding Patient Data team is recruiting a new policy/comms officer #datasaveslives”	@NMRPerrin; as above	37	3	138,791
8	“Register now for our Annual Scientific Meeting- Research in the Digital Age #DataSavesLives”	@SMHRN1; Scottish MH Research Network-supporting excellence in mental health studies as part of NHS Research Scotland	36	2	62,589
9	“New #INTEROPen board: an open collaboration of #interoperability networks to drive #OpenStandards in #health & #socialcare #DataSavesLives”	@INTEROPenAPI; Leading organizations supporting patients clinicians & new care models. Accelerating the delivery of #Interoperability #OpenStandards in health & social care	33	4	37,204
10	Not available. Tweet deleted by the user	Bio not available	31	N/A	N/A

^a^As of August 31, 2017.

^b^Group numbers cross-referenced with Table 3.

^c^Calculated as the sum of followers across all users who retweeted the original post. This method overestimates the total potential reach as it cannot account for the overlap of followers between users, and in any case, it is unlikely that all followers would view posts.

^d^N/A: not available.

### Who Tweets #datasaveslives?

There were 3649 unique Twitter users who posted or shared content, including #datasaveslives (Table 2). Approximately 1 in 10 (1573/13895, 11.32%) of all #datasaveslives tweets, and 1 in 6 of posts that used original text (421/2722, 15.46%), were by official supporters. The tweet type was significantly associated with an official supporter status; official supporters used posts with original text relatively more often than others (26.76% vs 18.67%; χ_1_^2^=57.5; *P*<.001).

Among the 3649 users who posted or shared #datasaveslives at any time during the time window observed, 64.87% (2367/3649) did so only once (range 1-455). Users who tweeted 10 times or more accounted for just 4.88% (178/3649) of users, yet produced 54.33% (7549/13,895) of tweets; 16 users tweeted 100 times or more. This included 5 of the 6 official supporters, plus the accounts of affiliated organizations and projects. A total of 13 of the 16 accounts were associated with groups. In addition to official supporter organizations, these included health charities, professional membership organizations, event organizers, and projects. Notably, one of these frequent tweeters was a patient advocate and campaigner (n=102 tweets).

**Table 2:** Tweet frequency by tweet type and user type.

Tweet type		Tweet frequency by user type, n (%)		Total tweets (n=13,895), n (%)	Total unique users (n=3649), n (%)^a^
		Official supporter (n=1573)	Other (n=12,322)		
**Original posts**					
	Original text	421 (26.76)	2301 (18.67)	2722 (19.60)	613 (16.80)
	Quote	243 (15.44)	1210 (9.82)	1453 (10.46)	551 (15.10)
**Shares**					
	Retweet	909 (57.79)	8811 (71.51)	9720 (69.95)	3157 (86.52)

^a^Owing to the overlap between users who use posts and shares, this column does not add up to 100%.

### How Were Tweets Shared Between Users?

**Figure 2:**
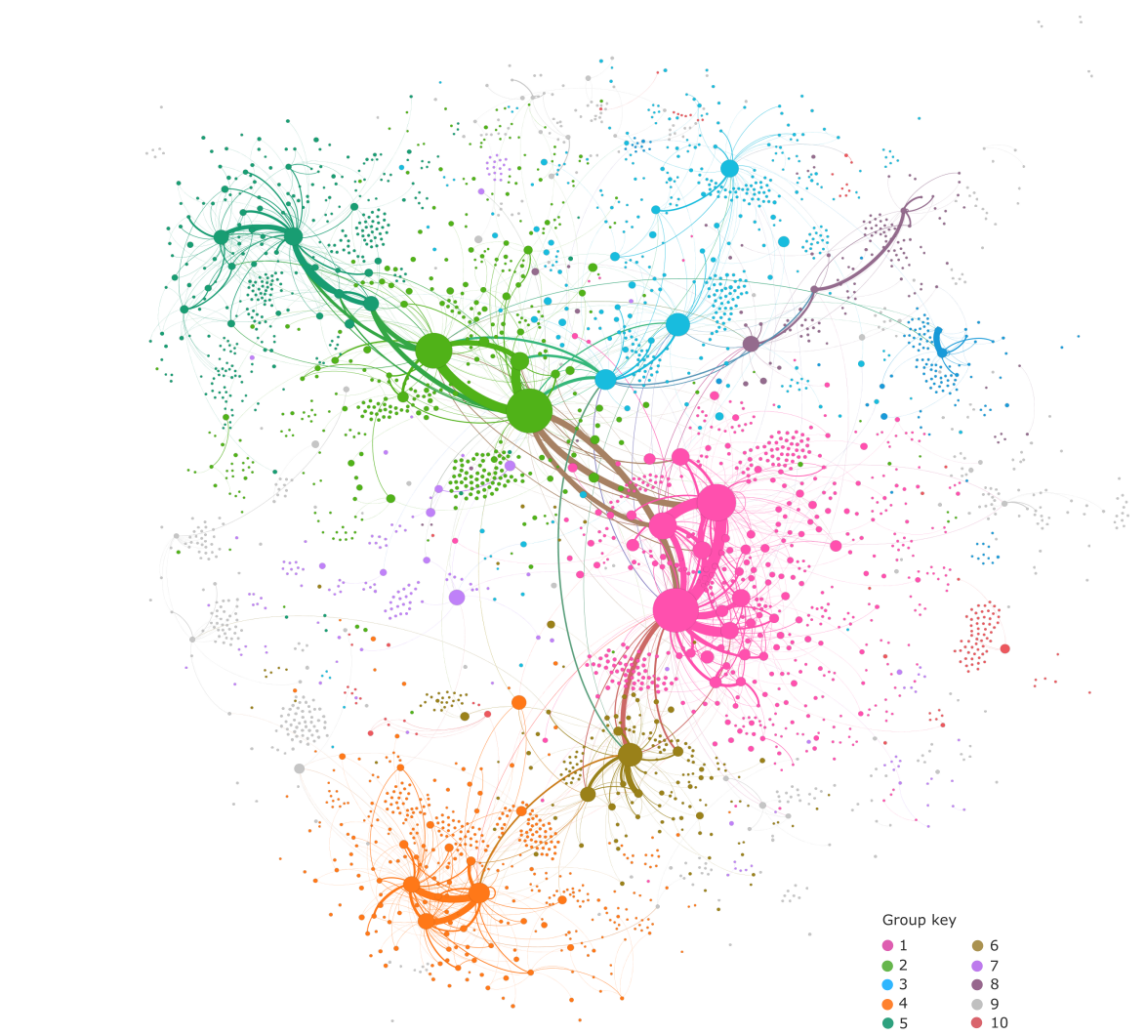
Retweet network graph showing relationships between users who tweet and retweet #datasaveslives.

We visualized retweet relationships between Twitter users as an undirected network graph (Figure 2). Retweet connections were created when a user shared content by another user that included the hashtag. The analysis of retweets (n=9720) generated a network of 3392 users and 5749 unique connections between pairs of users (average degree=3.39; average path length=4.02; diameter=12).

Cluster analysis using the Louvain method of community detection revealed 98 relatively well-connected groups (modularity=0.684). These were arranged in *hub and spoke* structures, with smaller numbers of relatively more tightly connected users at the center of each group. The 5 largest clusters or groups contained 60.70% (2059/3392) of users in the network; 69 groups were very small, containing 5 or fewer users.

We examined the size, users, and words used in user bios for the largest 10 groups yielded by the cluster analysis (Table 3). The largest 2 groups (1 and 2) included all 6 official supporter accounts and were closely connected. Groups 1 and 2 shared similar vocabulary, both for tweets and user bios (eg, *health*, *research*, and *university*).

**Table 3:** Users in the #datasaveslives retweet user network by group.

Group	Users, n (%)	Most influential organizational user accounts (eigenvector centrality)^a^	Top 5 words used in user bios (n)	Top 5 words used in user tweets (n)
1	533 (15.71)	@CHCNorth (0.99), @HeRC_Farr (0.82), and The _NHSA (0.35)	Health (154), Manchester (71), research (67), university (64), and science (43)	Data (198), health (189), great (80), #iforh2017 (70) and research (53)
2	405 (11.94)	@FarrInstitute (1.0), @FarrScotland (0.79), and @FarrCIPHER (0.37)	Health (163), research (154), data (59), university (40), and public (39)	Data (143), health (96), research (51), #iforh2017 (46), and case (37)
3	399 (11.76)	@Patient_Data (0.43), @AMRC (0.36), and @wellcometrust (0.20)	Research (112), health (98), views (82), policy (44), and care (38)	Data (145), health (122), patient (60), information (57), and using (50).
4	390 (11.50)	@InteropSummit (0.33), @INTEROPenAPI (0.33), and @oht_uk (0.14)	Health (100), views (77), care (72), healthcare (44), and NHS^b^ (40)	#interopsummit (110), #interoperability (53), #interopwarrior (38), care (36), and data (35)
5	332 (9.79)	@cancerchallscot (0.37), @IHDPscot (0.30), and @ProductForge (0.29)	Health (60), Scotland (51), care (38), data (37), and cancer (36)	#cancerdatadive (73), cancer (61), data (58), great (29), and #hackathon (27)
6	181 (5.34)	@GreatNorthCare (0.50) and @AHSN_NENC (0.19)	NHS (34), health (33), clinical (22), care (21), and director (17)	NHS (34), health (33), views (32), clinical (22), and care (21)
7	151 (4.45)	@NHSDigital (0.31), @DeptHealthPress (0.16), and @Soc_Endo (0.13)	Health (26), digital (22), care (15), research (14), and NHS (13)	Views (36), health (26), digital (22), care (15), and research (14)
8	127 (3.74)	@useMYdata (0.32), @DNADigest (0.10), and @abcdiagnosis (0.09)	Cancer (54), breast (29), research (27), health (18), and advocate (13)	Cancer (54), breast (29), research (27), health (18), and views (17)
9	117 (3.45)	@UoLCardioEpi (0.18), @LabKey (0.04), and @HealthSciYork (0.02)	Research (24), care (4), health (14), cardiovascular (13), and university (10)	Research (24), care (14), health (14), cardiovascular (13), and views (13)
10	110 (3.24)	@NHSEngland (0.17), @MedineGov (0.10), and @CURE_ScHARR (0.04)	Health (23), care (16), views (13), NHS (12), research (9), and healthcare (9)	Health (23), care (16), views (13), NHS (12), and research (9)

^a^Maximum of 3 users in the top 10 accounts.

^b^NHS: National Health Service.

Closer examination indicated some distinctions between groups 1 and 2. Group 1 users were more strongly affiliated with Northern England (particularly Manchester), whereas group 2 users frequently referenced places, organizations, and events located in Scotland. Group 1 was closely connected with group 5, which had a distinct topic focus on cancer data. Group 2 showed a stronger connection with group 6, which was associated with major medical records information technology (IT) projects based in the North East of England. Group 6 was, in turn, connected with group 4, populated by National Health Service (NHS) staff and delegates of a major health care IT conference (indicated by *#interopsummit*).

Group 3 indicated connections with both groups 1 and 2, and included users with connections to the NHS, health care policy, and major charities. Commonly used words in this group suggested a more applied focus among users (eg, *policy* and *care*). Group 3 was also loosely connected to group 8, distinctly notable for comprising users who self-identified as patients, carers, and advocates.

### What Did People Use #datasaveslives to Tweet About?

The thematic analysis of tweet content yielded 4 key ways in which #datasaveslives was used: to share information and updates, for reporting and discussion at events, to show support for data sharing, and as a call to action. Although themes have been described separately for clarity, in practice there was substantial overlap, with the same tweets often being classified under multiple themes (Table 4).

**Table 4:** Examples of tweets with overlapping themes.

Theme^a^				Example tweets	
A	B	C	D		
✓^b^	✓	N/A^c^	N/A		“Today is **#**WorldHealthDay - Find out how we work to improve health & care for patients & public here: [link to website] #datasaveslives” [@FarrInstitute]
✓	N/A	✓	N/A		“Interesting paper from @[usernames] calls for clarity on conflicting data sharing guidance [link to website] #datasaveslives” [@Patient_Data]
✓	N/A	N/A	N/A		“We are using patient data to implement learning health systems across the #North. Find out more: [link to website] #datasaveslives” [@AMRC]
N/A	✓	✓	N/A		“The Farr Institute discusses importance of patient data at House of Commons event #APPGMedResearch #datasaveslives [link to website]” [@FarrInstitute]
N/A	✓	N/A	N/A		“Thank you to all of our speakers today, to find out more about their work follow @UoLCardioEpi #datasaveslives #LIDASeminar” [@LIDA_UK]
N/A	N/A	✓	✓		“Everybody should be able to find out how patient data is used. Read our case studies on how #datasaveslives… [link to website]” [@Patient_Data]
✓	✓	✓	N/A		“We believe #DataSavesLives! As do #interopsummit lecturers VIDEOS of Day 2 lectures on @YouTube [link to website] #interoperability” [@InteropSummit]
N/A	✓	✓	✓		“If you're at #IforH2017 don't forget to take a selfie with #datasaveslives at our stall (12) - just like [first name] from @[username] [photo]” [@FarrScotland]
✓	N/A	✓	✓		“Help contribute to the latest inquiry by @LordsSTCom into the #LifeSciences #IndustrialStrategy and highlight that #datasaveslives [link to website]” [@AMRC]

^a^Qualitative theme descriptions: A, to index and share information; B, for reporting and discussion at events; C, to show support for data sharing; and D, as a call to action.

^b^Data are applicable to themes.

^c^N/A: not applicable.

#### To Index and Share Information

The most common types of posts featuring #datasaveslives, particularly by official supporters and members of groups 1, 2, and 3 (Figure 2 and Table 3), were tweets sharing information about users’ own projects, research findings, and news. These included announcements about new projects or funding, updates on progress, and sharing results from research. Although some tweets directly referenced peer-reviewed scientific literature by linking to journal publications, more often they were linked to less formal sources, including project websites, case studies, blogs, and videos:

Thanks to data we know that the smoking ban in Scotland has been a success [link to case study on website] #datasaveslives

@FarrScotland, Group 2

Highlights from Informatics for Health 2017 by @HeRC_Farr: Watch the video at [website link] #IforH2017 #datasaveslives

@FarrInstitute, Group 2

Twitter users also used #datasaveslives to highlight the work of others and signpost wider news and policy developments in areas relevant to health data science. These included news stories published by universities, health service organizations, professional bodies, and reports in popular media, including the local and national press and television and radio programs:

BBC News - Artificial intelligence predicts when heart will fail [link to news report] #DataSavesLives

@EmpowerD4H, Group 13

In the vast majority of cases, references for data sharing were positive or at least neutral; occasionally, however, there was evidence of more critical commentary about certain uses of health data:

Check out how @ukhomeoffice using health information is denying patients healthcare [link to news story] #DataSavesLives until it doesn't

@einsteinsattic, Group 2

Among tweets in this category, hyperlinks to other websites were very common; indeed, a subgroup of tweets were identified that included a hyperlink and the hashtag, indicating the use of #datasaveslives as purely an index function. This was mainly used by official supporters.

#### For Reporting and Discussion at Events

Frequently, #datasaveslives was used to tag tweets related to events, including conferences, meetings, and public engagement activities. Tweets included the promotion of forthcoming events, discussion of past events, or even live reporting and commentary about events, talks, and discussions that were currently underway. In the case of larger events, such as conferences, #datasaveslives frequently appeared alongside other official event hashtags (eg, #iforh2017, #interopsummit). Images of slides, presenters, delegates, visitors, and stalls were commonly included alongside the text:

Looking forward to meetings workshops and exciting stuff at @ExpoNHS tmrw #datasaveslives#nhs

@ruthlady, Group 1

#### To Show Support for Data Sharing

One further use of #datasaveslives was to demonstrate personal support for sharing health data in general or backing the #datasaveslives campaign itself. A total of 26 users included the text #datasaveslives within their Twitter bio. Many tweets of this type included images of individuals or groups at events pointedly posing with eye-catching placards, badges, or clothing featuring the hashtag:

Thanks for coming to chat wear your badge with pride!

@FarrInstitute, Group 1

Some tweets included a positive statement about reasons for supporting data sharing, either within the tweet or written on placards pictured in the tweet. The reasons referenced included sharing health data for research, sharing data as part of routine health care, or sharing data as part of larger projects that combined elements of both. Some tweets within this category signposted wider evidence supporting data sharing, such as collections of case studies where health data had been used for patient benefit. These were especially common among groups 4 and 5. Some drew on first-hand experiences and opinions:

For more examples of how #datasaveslives in mental health read this @MQmentalhealth blog. See our case studies [link to website]

@Patient_Data, Group 3

The type of treatment that I had depended so much on the data of patients who went before me’ - patient advocate - #datasaveslives

@useMYdata, Group 8

#### As a Call to Action

We also identified a category of tweets that were used to make requests for others to act, participate, or respond in some manner. Commonly, these included advertisements to register for or submit papers to future events, participate in research studies, visit exhibition stands at conferences, or apply for jobs. There were also requests to provide feedback, opinions, or information:

We're inviting applications for a 2yr Clinical Research Fellow to study for an MD. Cardiology trainees please. #heartattack #datasaveslives

@UoLCardioEpi, Group 9

Help guide our consent modelling framework: happy to share a copy of your care org's consent forms? TY/please DM #datasaveslives #ontology

@GreatNorthCare, Group 6

## Discussion

### Principal Findings

This study investigated how a dedicated hashtag was used to promote the reuse of health data for research purposes and public benefit, how often, and by whom. Originally launched by the Farr Institute for Health Informatics Research, #datasaveslives came to be adopted by several distinct, diverse, yet interconnected groups in the United Kingdom with shared interests in health informatics, policy, and research. Our findings suggest that reasons for tweeting #datasaveslives evolved beyond the original objective of indexing information to a broader range of purposes, including event reporting, encouraging participation and action, and showing support for sharing health data.

### Comparisons With Previous Work

Among the wider range of communities who shared content tagged with #datasaveslives, we detected 2 communities in particular who were research-focused, geographically distinct, and strongly interconnected. These were, in turn, connected with distinct professional communities with wider interests—some with access to sizable networks, funding, and influence—including government departments, the NHS, policy makers, patient advocates, and major charities. Our findings fit with the wider literature, which indicates that scientists can use Twitter not only to communicate with each other but also to engage broader audiences, including policy makers and the public [3,6].

One of the initial, more obvious uses of the hashtag was to index information about the use of health data as part of research and innovation, and make it more readily retrievable to a wider, not exclusively scientific, audience. Moreover, people also used the hashtag to publicly demonstrate support for data sharing and each other. This is compatible with the wider literature, which suggests that academics use hashtags to categorize information [26] and encourage interaction and community building [27-29]. These uses seem pertinent, given that our period of observation followed the high-profile failure of the care.data scheme, a major government initiative in England to share patient data [30]. Indeed, two of the most frequently shared tweets in our analysis concerned subsequent proposals to change government policy, addressing data security and consent [24,25]. Previous studies have shown how responses to care.data on Twitter attracted critical commentary [31], including from interest communities in politics, health care, and the media [32]. Before the observation period examined in this study, concerns had been raised about access to patient data by commercial companies, especially where these uses were perceived to be primarily motivated by profit rather than public benefit [30,33-35]. This study contrasts with these findings, showing how #datasaveslives was used in the wake of public backlash to care.data to spread mainly positive messages about data use and reuse, and to increase transparency, demonstrate solidarity, and provide supportive networks among health, data, and IT professionals.

In declaring an intent to promote the reuse of health data for research purposes, the #datasaveslives campaign could be regarded as a behavioral intervention of sorts, encouraging credible users to endorse and share supportive messages. As with other behavioral interventions conducted via social media, attention should be directed toward identifying the active ingredients of interventions [36]. Our thematic analyses of tweet content revealed 2 noteworthy and interrelated strategies used as components to achieve campaign aims. First, #datasaveslives was used at events frequented by influential communities, generating spikes in activity generated by commentary about the proceedings of meetings and events in real time. So-called *live-tweeting* has become more common at scientific conferences and has the advantage of increasing transparency and rapidly disseminating information among a far larger audience over and above those who physically attend [37,38]. Using #datasaveslives, either alone or in addition to more specific conference hashtags, might have amplified the reach of information while avoiding the limited audience and *shelf-life* of more niche conference hashtags.

Second, offline activities at events were used to drive the generation of web-based multimedia content; events were used as photo opportunities for individuals willing to publicly endorse #datasaveslives, leveraged by attractive branded physical merchandise. Drawing on evidence from previous studies, which have identified health behavior change techniques particular to social media, reviewed by Simeon et al [36], these photo opportunities might be framed as *virtual rewards*, in turn encouraging further *overt endorsements* in the form of likes, retweets, and comments. Indeed, similar social media strategies have been used in both the health sciences and the corporate sectors, such as identifying target communities, gaining support from credible and/or influential users, developing engaging multimedia content, updating content regularly, improving the visual presentation of content, and encouraging participation via small concrete actions [16,39,40].

### Strengths and Limitations

This study benefits from the analysis of a near-complete sample of #datasaveslives public tweets for an active year during the campaign. Nonetheless, we could not have captured all mentions and uses of #datasaveslives during this period. Private and previously deleted tweets were excluded. Owing to the limited use of other social media platforms by official supporters, our analysis only considered Twitter posts tagged with the keyword #datasaveslives. It is notable that other important public health outreach campaigns—including during outbreaks [41], as part of science communication [16] and to promote health behavior change [36]—have commonly used a wider range of social media platforms, particularly Facebook. The content, strategies, and communities observed in this study may be specific to Twitter and should not be generalized to other social media or content-sharing platforms. Furthermore, the network analysis was limited to retweets; we did not capture other types of engagement, such as follower networks, or use directed networks, as done by other studies [32]. Thus, certain nuances of information flow may have been lost, indicating influential relationships. Demographic data about users were not made available by Twitter for analysis, limiting our understanding of sample characteristics. Finally, we accept that we were unable to quantify, much less characterize, the much wider audience who saw, read, or otherwise engaged with tweets, in particular patients and members of the wider public not connected to organizations.

### Future Research

The health data science community has stated a vision to be team-based, transparent, and inclusive, seeking involvement from a wide range of interdisciplinary stakeholders, including patients and the public [42]. Future research would benefit from examining how the use and users of #datasaveslives have changed over time and suitable ways of determining the overall impact of varying strategies to engage key communities, such as members of the public. Using such opportunities for social media to contribute toward building networks and engaging in dialog in open forums would seem eminently compatible with this vision.

### Conclusions

The rise of social media has provided unprecedented opportunities for academic organizations and individual scientists to communicate with a much wider range of stakeholders than ever before, including the public. This study shows how a simple hashtag campaign on Twitter was used to disseminate credible scientific information and increase the visibility of research activities, with evidence to suggest this supported community building and bridging practices among interdisciplinary sectors allied to health data science.

Our findings are of interest to a variety of stakeholders who share an interest in supporting the reuse of health data for public benefit. By revealing the different communities who share such interests, analyzing content thematically, and demonstrating how information flows between them, our findings can be used to better understand the mechanisms underpinning stakeholder engagement campaigns conducted on social media and how to optimize these further.

## Abbreviations

CHC: Connected Health Cities
IT: information technology
NHS: National Health Service
